# An integrated insight into the response of sedimentary microbial communities to heavy metal contamination

**DOI:** 10.1038/srep14266

**Published:** 2015-09-22

**Authors:** Huaqun Yin, Jiaojiao Niu, Youhua Ren, Jing Cong, Xiaoxia Zhang, Fenliang Fan, Yunhua Xiao, Xian Zhang, Jie Deng, Ming Xie, Zhili He, Jizhong Zhou, Yili Liang, Xueduan Liu

**Affiliations:** 1 School of Minerals Processing and Bioengineering, Central South University, Changsha 410083, China; 2 Key laboratory of Biometallurgy, Ministry of Education, Changsha 410083, China; 3 College of Food Science and Technology, Hunan Agricultural University, Changsha 410083, China; 4 Key Laboratory of Microbial Resources Collection and Preservation, Ministry of Agriculture, Beijing 100081, China; 5 Key Laboratory of Plant Nutrition and Fertilizer, Beijing 100081, China; 6 Institute of Agricultural Resources and Regional Planning, Chinese Academy of Agricultural Sciences, Beijing 100081, China; 7 School of Environment, Tsinghua University, Beijing 100084, China; 8 Institute for Environmental Genomics and Department of Botany and Microbiology, University of Oklahoma, Norman 73019, USA; 9 Earth Sciences Division, Lawrence Berkeley National Laboratory, Berkeley 94710, USA

## Abstract

Response of biological communities to environmental stresses is a critical issue in ecology, but how microbial communities shift across heavy metal gradients remain unclear. To explore the microbial response to heavy metal contamination (e.g., Cr, Mn, Zn), the composition, structure and functional potential of sedimentary microbial community were investigated by sequencing of 16S rRNA gene amplicons and a functional gene microarray. Analysis of 16S rRNA sequences revealed that the composition and structure of sedimentary microbial communities changed significantly across a gradient of heavy metal contamination, and the relative abundances were higher for *Firmicutes*, *Chloroflexi* and *Crenarchaeota*, but lower for *Proteobacteria* and *Actinobacteria* in highly contaminated samples. Also, molecular ecological network analysis of sequencing data indicated that their possible interactions might be enhanced in highly contaminated communities. Correspondently, key functional genes involved in metal homeostasis (e.g., *chrR*, *metC*, *merB*), carbon metabolism, and organic remediation showed a higher abundance in highly contaminated samples, indicating that bacterial communities in contaminated areas may modulate their energy consumption and organic remediation ability. This study indicated that the sedimentary indigenous microbial community may shift the composition and structure as well as function priority and interaction network to increase their adaptability and/or resistance to environmental contamination.

How microbial communities respond to environmental changes is a key issue in ecology. In recent years, industrial activities have a significant influence on the environment. Especially, heavy metals such as Hg, Cr, Pb, Mn, and As have induced serious diseases or even death of organisms through contaminated waters or soils, although heavy metals in trace amount are beneficial even significant to organisms^1^,^2^,^3^. A series of studies of bioremediation have been conducted and many metal resistance genes in microbes have been identified^4^. To date, such studies have been focused on functional and phylogenetical analysis of microbial response to environmental contamination. For example, in highly contaminated sites, an overall lower gene diversity but higher abundance for specific functional genes, such as heavy metal homeostasis genes and sulfate-reducing genes were observed^1^,^5^. Also a previous study revealed that the greatest species diversity appeared in the moderately contaminated sedimentary samples, and the dominant groups included *α-Proteobacteria*, *β-Proteobacteria* and *Firmicutes*^6^. Interestingly, Chodak *et al.*^7^ reported that the effect of heavy metal contamination on the structure of soil bacteria measured by pyrosequencing was not observed as the abundance of many phyla remained unchanged.

The recent development of new technologies provides opportunities to explore those complex microbial communities and their response mechanisms to heavy metal contamination. Particularly, high-throughput sequencing and functional gene arrays are powerful tools to study the functional diversity, composition, structure and metabolic potential of microbial communities^8^. For instance, GeoChip 5S (GeoChip 5.0, small version), containing about 60,000 probes in biogeochemical cycling of carbon (C), nitrogen (N), sulfur (S) and phosphorus (P), has been applied to analyze soil microbial communities^9^. Also, metagenomic sequencing provides us an opportunity to explore the complex and primarily uncultured microbial communities. Recently, Illumina MiSeq sequencing of 16S rRNA genes has been used to explore the shifts of tundra bacterial and archaeal communities along a permafrost thaw gradient^10^.

Xiangjiang River is the tributary of Yangtze River, the valley area of 9.46 square kilometers, located in central south of China. It provides drinking water for about 20 millions of people, irrigation water for 13,200 square kilometers of cultivated lands, and also important to fishery and shipping industry^11^. In past 30 years, the river has been heavily contaminated by heavy metals (e.g., Hg, Pb, As)^12^, and caused many serious pollution incidents^11^,^12^. However, it remains unclear how sedimentary microbial communities respond to heavy metal contamination, and what mechanisms microorganisms may use to adapt to contaminated environments.

In this study, we hypothesized that (i) the phylogenetic diversity and structure of microbial community would shift under heavy metal contamination; (ii) heavy metals would affect microbial network interactions among different phylogenetic groups; and (iii) the functional composition and structure of microbial communities would differ across a gradient of heavy metal contamination. To test these hypotheses and explore their adaption mechanism, 16 sedimentary samples were taken from four sites in the Xiangjiang River and analyzed by GeoChip 5.0 and sequencing of 16S rRNA gene amplicons. The study may provide us an integrated insight into the response of microbial communities to heavy metal contamination.

## Results

### Sediment geochemical properties

ICP analysis of heavy metals revealed the concentrations of Hg, As, Co, Cd, Cr, Ni, Pb, Cu, Mn and Zn as well as the amount of total nitrogen and total carbon (Table S2). The concentrations of Hg, As, Cd, Cr, Ni, Pb, Cu, Mn and Zn were severely higher than the maximum concentrations allowed in the river system. Take Cr as an example, its amount was 591 ~ 805 times of the national standard values (0.1 ppm), indicating the river had been heavily contaminated. Detrended correspondence analysis (DCA) of those heavy metal concentrations showed that 16 samples were clustered into two groups, namely H group (A1, A2, A3, A4, D1, D2, D3, D4), and L group (B1, B2, B3, B4, C1, C2, C3, C4) (Figure S1). The concentrations of all geochemical attributes (e.g., Mn, Zn) in H group were significantly (p < 0.05) higher than that in L group, except for Pb.

### Phylogenetic composition and structure of sedimentary microbial communities

A total of 321,671 high quality 16S rRNA gene sequences were obtained for all 16 samples, and they were resampled with 13,000 sequences per sample, which were clustered into 3,191 OTUs. The rarefaction curve showed that our sequencing efforts were enough for this study as the number of OTUs were almost saturated (Figure S2). Both Shannon diversity and Pielou evenness indices showed no significant difference between two groups of samples (Table S3). However, the structure of sedimentary microbial community was significantly different (p < 0.1) between those two groups demonstrated by dissimilarity tests based on sequencing data.

Analysis of 16S rRNA gene sequences showed that the community composition was apparently different between two groups (Figure S3). 746 OTUs were shared by both H and L groups, and these shared OTUs belonged mainly to *Firmicutes* and *Proteobacteria*. Both H and L communities were mainly composed of *Proteobacteria* (35.79% for H and 52.16% for L, respectively), followed by *Firmicutes* (19.81% and 40.78%) (Figure S3). Other major phyla for both communities were *Bacteroidetes* (5.94%, 9.91%), *Acidobacteria* (6.06%, 5.34%) and *Actinobacteria* (3.23%, 5.35%). In addition, 1%–5% OTUs could not be classified into any known phylogenetic groups. In H group, the most abundant genera were *Fusibacter* (27.56%) and *Proteiniclasticum* (6.66%), while *Fusibacter* and *Janthinobacterium* accounted for 14.24% and 10.79% of all sequences in L group, respectively (Figure S3).

**Figure 1:**
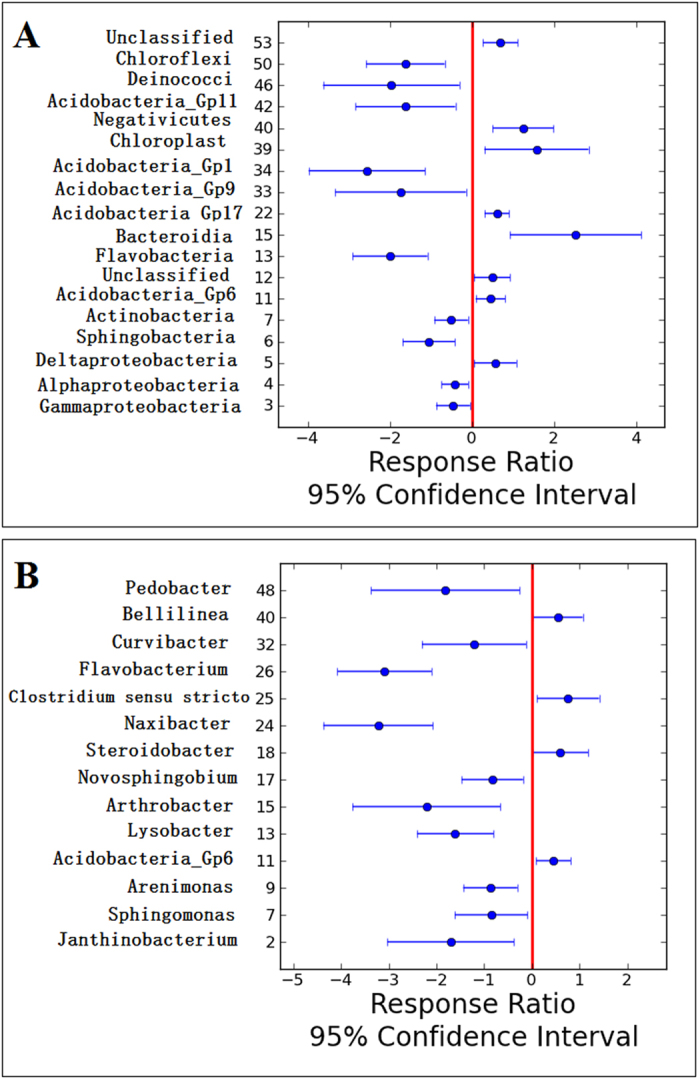
Response ratio of bacterial relative abundance of H group to L group at class level (A) and genus level (B) with 95% confidence.

To reveal how the sedimentary microbial community shifts across the metal concentration gradient, response ratio analysis was conducted, showing that there were higher percentage of *Firmicutes*, *Chloroflexi* and *Crenarchaeota*, and lower percentage of *Proteobacteria*, *Actinobacteria* in heavily contaminated sites (H group) at the phylum level. At the class level, *Deltaproteobacteria*, *Acidobacteria_Gp6*, *Gp17* and *Bacteroidia* were more abundant in H group, whereas *Gammaproteobacteria*, *Alphaproteobacteria* and *Sphingobacteria* were less abundant in H group (Fig. 1a). At the genus level, *Acidobacteria_6* and *Steroidobacter* were more abundant in H group, while the relative abundance of *Janthinobacterium*, *Sphingomonas* and *Arenimonas* were higher in L group (Fig. 1b).

### Effects of heavy metals on the co-occurrence of microorganisms

To understand the co-occurrence of microbial populations in both H and L microbial communities, OTU data of 16S rRNA sequences were used to construct molecular ecological networks (MENs) for H and L groups by RMT-based network approach. Major topological properties of two empirical MENs (H-MEN and L-MEN) of microbial communities showed that, with the same threshold (0.900), there were a lot more nodes and links in H group (394 nodes, 1609 links) than in L group (183 nodes, 406 links) (Table 1). The degree distributions in both constructed MENs well fitted the power law model as linear correlations were 0.873 and 0.824, respectively, although the degrees of distribution also fitted well with two other models (truncated power law and exponential power law) (Figure S4). For the average path distance, H-MEN had the value of 3.598, less than 4.449 in L-MEN, suggesting that H-MEN might more closely connected than L-MEN. The same tendency was also seen from Figure S5.

Furthermore, eigengene analysis^13^,^14^ was performed to reveal the higher-order organization of the constructed MENs. In this analysis, each module is summarized through singular value decomposition analysis with a single representative abundance profile, which is referred to as the module eigengene. Our results showed that module eigengene explained 37–70% of the variances in relative OTU abundance across different samples in H group and 43–79% of that in L group. Most of the eigengenes (12/14) could explain more than 50% of the variations observed. The results suggest that these eigengenes relatively well represented the changes. Module membership was evaluated to determine the extent to which an OTU was associated with a module. Most of the OTUs had significant module memberships with their respective modules. For example, module E5 in H group had 24 OTUs derived from *Bacteroidetes*, *Chloroflexi*, *Proteobacteria*, *Acidobacteria*, *Firmicutes* and *Actinobacteria*. Its eigengene could explain 59% of all the variations (Figure S6). Additionally, the relationship between microbial network modules and sediment properties were analyzed with Mantel tests. It was found that Hg, Pb, Zn and C were significantly (r_M_ < −0.6, p < 0.1) correlated with module E5 in H group.

**Figure 2:**
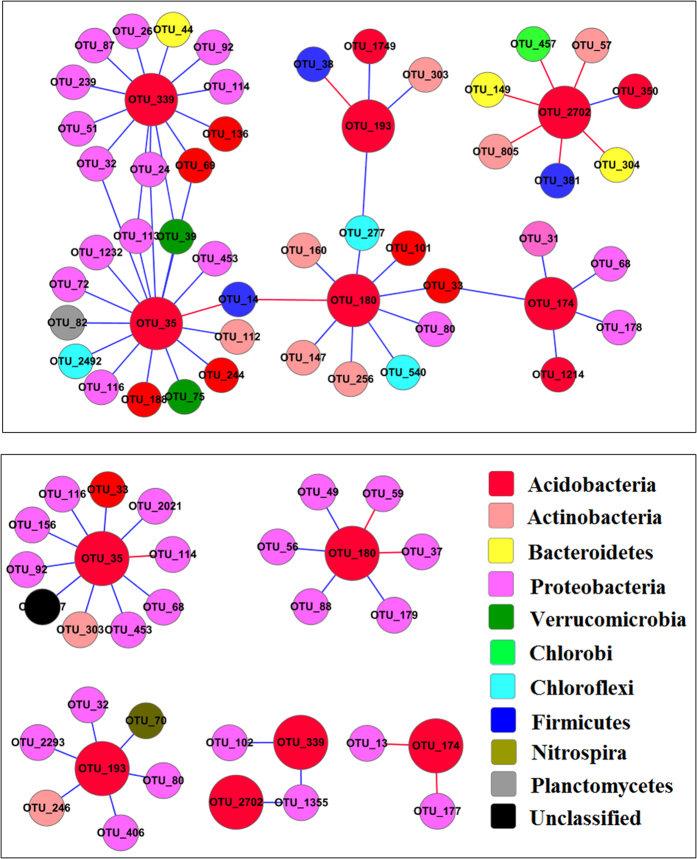
Effects of heavy metals on the network interactions of *Acidobacteria_Gp6*. (**A**) Network interactions of the top six OTUs of *Acidobacteria_Gp6* with the highest connectivities in H group. (**B**) Network interactions of the corresponding OTUs of *Acidobacteria_Gp6* in L group.

**Figure 3:**
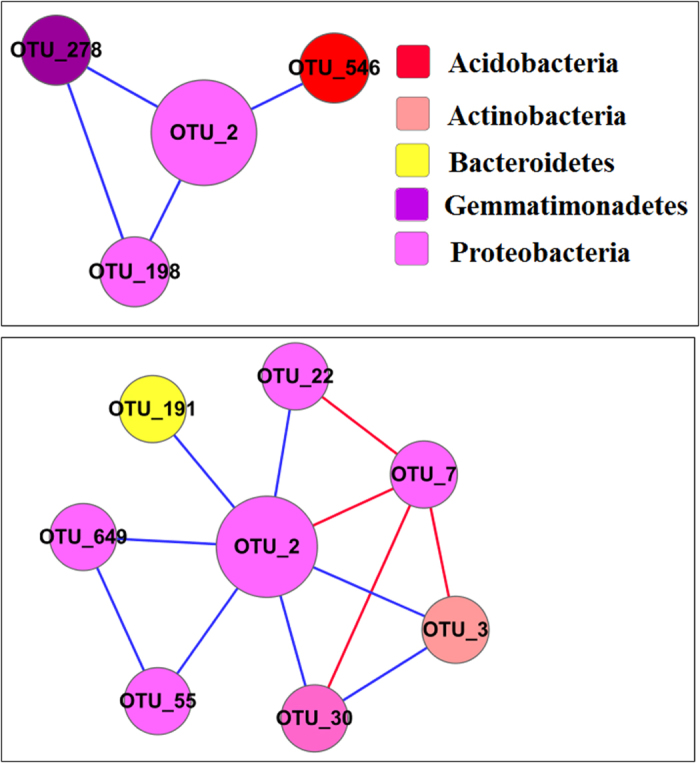
Effect of heavy metals on the network interactions of the only OUT of *Janthinobacterium* in H group (A) and L group (B).

Also, we constructed sub-networks for *Acidobacteria_Gp6* and *Janthinobacterium* to further analyze their possible interactions with other microorganisms. We analyzed the network interactions of *Acidobacteria_Gp6* (Fig. 2) with the highest connections in order to explore the possible interaction between *Acidobacteria_Gp6* and other microbes and the mechanism by which the *Acidobacteria_Gp6* adapt to the heavy metal contaminated environments. The top six *Acidobacteria_Gp6* OTUs in H group had more complex interactions than their corresponding OTUs in L group, evidenced by more nodes and links. They had no connections with *Firmicutes* or *Chloroflexi* in L-MEN, but three positive links with *Chloroflexi* and three negative links with *Firmicutes* in H-MEN. Similarly, we constructed a sub-network of *Janthinobacterium* in order to explore why the relative abundance of *Janthinobacterium* decreased under heavy metal stress. Interestingly, there was only one nodes (0.34% of all nodes) related to *Janthinobacterium* (Fig. 3), although it accounted for about 6.38% of all reads. The results were different from *Acidobacteria_Gp6*, which accounted for 1.54% of all reads but 7.11% of all nodes, suggesting that network could reflect how microorganisms use their strategies to survive under stress conditions. OTU2 was connected with seven nodes in L-MEN, but only three in H-MEN. The results may be interpreted that the interaction of *Janthinobacterium* with other bacteria was weakened by heavy metals.

### Overview of functional genes in response to heavy metal contamination

A total of 29,439 gene variants were detected. They included gene groups of C, N, P, and S cycling, metal homeostasis, organic remediation and secondary metabolism. Shannon index and Pielou evenness were all high in both groups (Table S3), suggesting that there were a variety of functional genes in the Xiangjiang River sediment, although it was heavily contaminated by heavy metals. Also, the functional structure of sedimentary microbial communities shifted under heavy metal conditions. The result of dissimilarity test showed that most of the functional gene groups in H group were significantly (p < 0.1) different from samples in L group (Table S5), except for genes involved in secondary metabolism and virulence. The results suggested that the functional composition and structure of the microbial communities significantly changed in the heavy metal contaminated environment.

### Effects of heavy metals on key functional genes and processes

To further understand the effect of heavy metals on specific functional processes of soil microbial communities, key genes involved in C, N, S and P cycling were examined. Generally, 54 gene families had higher intensity in H group than in L group (Table S6a), including 21 genes involved in carbon cycling, four in metal homeostasis, three in nitrogen metabolism (*nasA* for assimilatory N reduction), 24 in organic remediation, and two in sulfur metabolism. In contrast, 51 genes showed lower intensity in L group (Table S6b), including 17 in carbon cycling, five in nitrogen metabolism, 23 in organic remediation, two in sulfur metabolism (*dsrA* and *dsrB* for sulfite reduction), one in phosphorus metabolism (*ppk* for polyphosphate synthesis), and three in virulence (*ben_bcla*, *fosx* for antibiotic resistance).

#### Metal homeostasis genes

**Figure 4:**
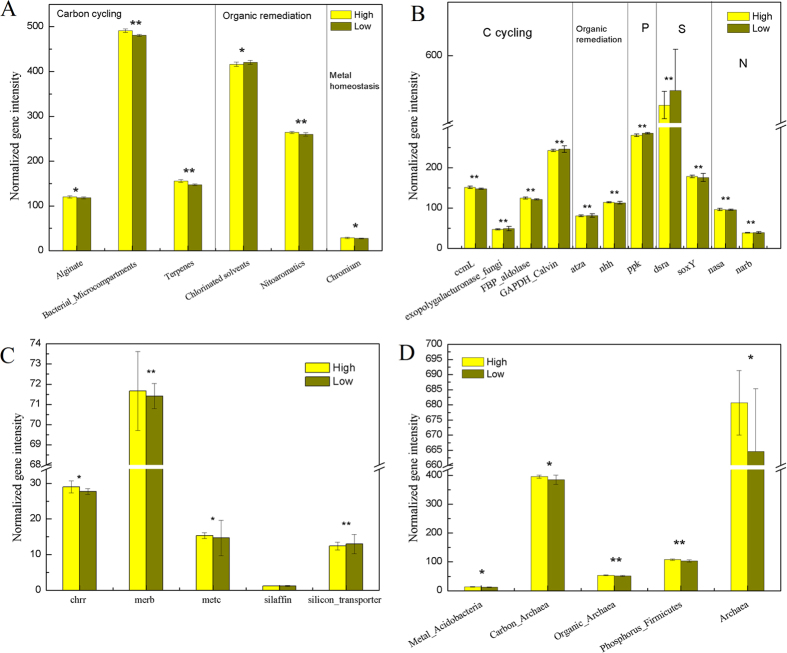
Normalized abundance of genes (genes groups) in each group. (**A**) Normalized gene intensity of each sub-group, including alginate, bacterial microcompartments, terpenes, chlorinated solvent, nitoaromatics and chromium. (**B**) Normalized intensity of each gene in groups of carbon cycling, organic remediation metabolism, phosphorus cycling, sulfur cycling, and nitrogen cycling. (**C**) Normalized intensity of metal homeostasis genes. (**D**) Normalized intensity of functional gene groups derived from specific microbial phylum (or domain).

A total of 1958 and 2133 probes had positive signals in H group and L group, respectively, which were involved in As, Cu, Hg, Si, Te and Cr resistance. At the level of gene family, only the intensity of chromium resistance related genes increased significantly (p < 0.1) in H group (Fig. 4a). Notably, four metal homeostasis genes showed differences between H and L groups, *chrR* (chromium detoxification), *metC* (mercury detoxification), *merB* (mercury detoxification), and *silaffin* gene (siliconbiosynthesis) (Fig. 4c). And their signal intensities were all significantly (p < 0.1) higher in H group than in L group. The results suggested that heavy metals increased the abundance of metal homeostasis genes so that their associated populations of sedimentary microbial communities might adapt to the environment.

#### C cycling genes

A total of 11,416 and 12,462 probes were present in H group and L group respectively, which were involved in C fixation, methane metabolism and C degradation. Dissimilarity tests showed that carbon metabolism related genes were different between two groups. Carbon degradation was important for microorganisms to get energy. For carbon degradation, eight genes conferring degradation of alginate, bacterial microcompartments, terpenes, pectin, vanillin/lignin, cutin, protein, lipids showed higher relative intensity in H group (Table S6), while sucrose, lignin, chitin, glucose, starch and cellulose degradation genes were less abundant in heavily contaminated sites. The results suggested that some carbon metabolisms were heavily affected by heavy metals while some functions were enhanced.

#### Organic remediation genes

About 6300 probes were involved in the degradation of aromatics, chlorinated solvent, halogenated compounds, herbicides and pesticide related compounds. The total signal intensity of the genes remained almost the same between the two groups. Nevertheless, genes involved in chlorinated solvents degradation were less abundant in H group, while nitoaromatics were more abundant (Fig. 4a). We also found that 24 genes showed higher intensity in heavily contaminated samples (Table S6), and 22 of them (e.g., *xylL*, *benD*, *nhh*) were related to aromatics degradation, while 23 genes, including those involved in the degradation of polycyclic aromatics, herbicides related compound and pesticides related compound, were less abundant in H group, but only 11 of them were involved in aromatics degradation.

### Relationships among the community structure, functional genes and sediment properties

To explore the effect of sediment properties on the microbial community structure, correlation analyses were performed by the Mental test (Table S4). Hg, Cu, Mn and Zn were generally correlated with the abundance of 16S rRNA gene sequences. The relative abundance of *Firmicutes* was positively correlated with Hg, Cd, Cu, Mn and Zn. And Cd had a significant (p < 0.05) effect on the abundance of Subdivision3, a class of *Verrucomicrobia* phylum, while Cd, Zn on *Actinobacteria*, and Hg, Cd, Cu, Zn on *Proteobacteria*.

At the aspect of functional genes, Co, Cd, Cr, Ni, Mn, Zn had significant effect on all gene groups, except for virulence (Table 2), and C and N were related to organic remediation genes only. At the gene family level, the signal intensity of many important genes showed significant correlations with the concentrations of Co, Cd, Cr, Ni, Cu, Mn and Zn (Table S7). Only *alkB* and *aryles*t, involved in organic remediation, were related to the amount of total C and N. These results suggest that the microbial community functional structure was correlated with heavy metal concentrations in the Xiangjiang River.

Moreover, the phylogenetic and functional data are mutually supported. Based on analysis of GeoChip data, we found: (i) for genes involved in metal homeostasis, the intensity of genes derived from *Acidobacteria* increased significantly in H group; (ii) the intensity of genes from *Firmicutes* increased for P metabolism; (iii) the total intensity of genes originated from Archaea increased in H group (Fig. 4d). The results comply with sequencing data that *Firmicutes*, Archaea and many *Acidobacteria* were more abundant in H group.

## Discussion

How microbial composition and structure shift under different contaminated environments is critical to reveal their adaptation mechanism to contamination. Our results showed that the sedimentary microbial community composition changed significantly under heavy metal conditions. The results generally supported our hypothesis that heavy metals would impact the sedimentary community structure. Although two groups of communities were mainly composed of *Proteobacteria* and *Firmicutes*, consistent with a previous study^6^, we demonstrated that there were more *Firmicutes*, *Chloroflexi* and *Crenarchaeota*, and less *Proteobacteria* and *Actinobacteria* in heavily contaminated samples (H group). Thus, it is supposed that the phyla *Firmicutes*, *Chloroflexi* and *Crenarchaeota* are highly resistant to heavy metals present in Xiangjiang River sediment, while *Proteobacteria* and *Actinobacteria* are less resistant or susceptible to heavy metals.

Of microbial populations highly resistant to heavy metals, the relative abundance of Archaea, mainly composed of *Crenarchaeota* in this study, increased in H group. *Crenarchaeota* were found in heavy metal contaminated acidic waters, indicating their high metal resistance^15^,^16^. Comparative genomic analyses in previous studies have shown that genes for metabolism, resistance, and detoxification of metals are widespread throughout the archaeal domain^17^, such as arsenic efflux and copper efflux. Also, there are researches about *Firmicutes*, which explain the high metal resistance of this phylum. Of them, a study of contaminated soils reported that metal-tolerant cultures were dominated by *Geobacter*-related *Deltaproteobacteria* and Gram-positive *Firmicutes* spp.^18^,^19^. A metal resistance mechanism may be related to Fe(III) reductive bacteria, which can tolerate millimolar concentrations of Cd, Cu, Ni, and Zn. *Acidobacteria GP6*, accounted for almost 30% of *Acidobacteria*, showed high abundance in this study, which may be related to their high metal resistance capability. *Acidobacteria GP6* have been found in uranium-contaminated sites^19^,^20^, indicating their high resistance to heavy metals. It is interesting that, as a phylum which includes many species capable of organic degradation^21^, *Chloroflexi* showed higher abundance in heavily contaminated sites, especially for the class of *Anaerolineae* and *Caldilineae*, consistent with previous study^6^. Commonly, *Anaerolineae* is often recognized as a large component of microbial communities in sludge wastewater treatment plants^22^, and has been known to be associated with anaerobic degradation of oil-related compounds. In this study, two metal resistance related genes, *arsC* and *terC*, detected in GeoChip were derived from *Anaerolineae*, and *arsM*, *mer*, *terC* were derived from *Caldilineae*, explaining their relatively higher abundance in H group.

On the contrary, bacteria that have relatively low resistance to heavy metals would account for lower abundance in the community, such as *Proteobacteria* and *Actinobacteria*. The similar study showed that a community was composed primarily of *γ-* and *β-Proteobacteria* in a heavy metal polluted groundwater^23^. In this study, about 62% of metal related genes were from *Proteobateria*. However, the abundance of *Proteobacteria* decreased in H group, especially γ-*proteobacteria* and α-*proteobacteria*. A possible reason is that some *Proteobacteria* were dramatically vulnerable to heavy metals. Taking *Janthinobacterium* as an example, it is an important genus of *Betaproteobacteria*^24^, and it was found to be susceptible to heavy metals, such as Ag, Cu, Hg, Pb and Ni^25^. In this study, the relative abundance of *Janthinobacterium* decreased significantly in highly contaminated samples, so did the ecological connections with other bacteria. Besides, *Actinobacteria* play an important role in the decomposition of organic materials and the production of secondary metabolites with very diverse physiology and few evidence shows that many of which are of high metal resistance.

Our ecological network analysis showed that heavy metals altered the network interactions among different microbial populations, supporting our hypothesis. In the network, positive interactions may reflect commonly preferred conditions or cooperative behaviors^26^, while negative interactions may reflect competitive behaviors, because organisms with similar traits may share similar niche requirements, which may result in them “sorting” into similar environments^27^, thus leading to competitive exclusion among organisms with very similar traits. Totally, heavy metals caused more links among OTUs, indicating that they tended to cooperate with each other to cope with the stress condition. Adversely, some bacteria who did not own the ability to compete with others would be weeded out^28^. Specifically, in H group, more *Acidobacteria GP6* were connected with other microorganisms, especially *Firmicute* and *Chloroflexi*, thus their abundance increased in heavily contaminated sites. On the contrary, the decreased ecological connections of *Janthinobacterium* with other microorganisms seemed to result in their decrease in relative abundance where there were more heavy metals in sediments. Coupled with evidence that *Firmicute* and *Chloroflexi* owned high metal resistance, it well explains why heavy metals selected for *Acidobacteria GP6* but against *Janthinobacterium*. Moreover, eigengene analysis revealed that heavy metals significantly altered the topological positions of individual OTUs, and Hg, Cr, Pb, Zn and C were significantly correlated with module formation. In a word, ecological network could well descript a whole scene of metagenomic data for us and well explain how the whole community structure shifts under stress conditions, suggesting relationships among microorganisms are modulated by environmental factors (heavy metals). But further experiments are needed to verify the theory.

However, it is out of our expectation that the alpha-diversity of microbial communities did not decrease significantly in H group, but even increased slightly. A pervious study showed that heavy metals would decrease the diversity of microbial community and functional genes, but we did not observe the same tendency either in microbial populations or functional genes^5^,^29^. Our observations comply with a similar study about heavy metal pollution in the Xiangjiang River, which showed that there was not a simple, negative relationship between heavy metal contamination and the genetic diversity of sediment microbial communities^6^. It was supposed that the microbes had already adapted to the polluted environments and could maintain their diversity by various of resistance mechanisms^30^. To date, it is generally accepted that simple systems are vulnerable to perturbations, so microbes need relatively high diversity to maintain their function^31^. However, current research results on this issue are still conflicting with each other.

Collectively, all the results above might provide some clues for us to unveil the molecular mechanism about how microbial communities shift to respond the contamination of heavy metals. Generally, microbial community shift is the result of adaptation of many kinds of bacteria, by taking various strategies. Some of resistant systems are widespread and serve in the basic defense of the cell against superfluous heavy metals, but some are highly specialized and occur only in a few bacteria. Specifically, cells have five basic mechanisms to improve their metal resistance, such as discharge toxic metals^32^,^33^. Moreover, there are also some other mechanisms that help microbial community survive under stress conditions, such as reductive precipitation, sulfate reduction and metal sulfide precipitation^34^. Our third hypothesis is that not only the metal resistance related genes but other functional genes would change under stress conditions, and the GeoChip data analysis complied with our hypothesis. Of course, metal homeostasis genes, like *chrR*, *metC* and *merB* were more abundant to realize chromium and mercury detoxification, and this was the key mechanism about how microbes adapted to polluted environments. Besides, we also found that gene groups involved in C, N, P, S, metal homeostasis and organic remediation showed significant difference between H and L samples, and these gene groups significantly correlated with the concentrations of Co, Cd, Cr, Ni, Mn and Zn, indicating the response of microbes to metal contamination is complex. In genes involved in sulfur metabolism, *soxY*, a gene conferring the function of sulfur oxidation, was more abundant in H group while *dsrA* for sulfite reduction was less abundant (Fig. 4b). Previous study showed that the *dsr* gene was most significantly correlated with pore water metals^5^, because sulfate reducing bacteria could reduce metal toxicity by precipitation or reduction. Our result is opposite with it, and the reason probably is that the correlation between *dsr* abundance and metals is metal type dependent. As for sulfur oxidation genes, it is well known that metals and S always coexist in extreme environments^35^, like sulfide minerals ore or acid mine drainage (AMD), which can explain the correlation between sulfur oxidation genes and metals.

Except for the basic finding that metal resistance related genes increased in H group to perform the key functions to survive in the polluted sediments, we also found some assistant and indirect metabolisms, which may impact the community more profoundly in a long term. In this study, we focused on the carbon metabolism and organic degradation, for many genes in these groups increased significantly in H group, indicating their importance in coping with metal contamination. First, previous studies show that the pathways of organic remediation and heavy metal pollution could share the same efflux pump and oxygenation complex in *Pseudomonas putida* KT2440^36^,^37^. So bacteria with capability of organic remediation may also have potential heavy metal resistance. It provides a possible way to explain why some organic remediation genes showed higher relative intensity in H group. Second, C source is not only necessary to maintain basic metabolism, but also can be used to absorb metals through complexation, reduction and volatilization of metal(loid)s^38^. For example, chitosan and lignin can be used to absorb Cr^39^,^40^. Our results revealed that metals and organic C were closely positively related, consistent with previous observations^41^. This study also indicated that microbes may give priority to degrade carbon source like Pectin, Vanillin/Lignin, Protein and Lipids, while the metabolism related to sucrose, lignin, chitin, starch, cellulose degradation were weaken. Heavy metals did have impact on substrate utilization pattern of microbial communities^42^,^43^. The reason may be that lignin and chitin were bound with metal ions so not available for microbial degradation.

Finally, we take module E5, which had high Phi value (59%) and significant correlations with environmental factors, as an example to illustrate how different microbial populations cooperate to survive in heavily contaminated environments. Basically, genera with metal resistance genes survived even prospered in heavily contaminated sites, such as OTU171 (*Legionella*) and OTU123 (*Rhodobacter*). Metal homeostasis genes, like *arsC*, *chrR*, *mer* and *terC* from these two genera were detected, and their intensity was higher in H group. Furthermore, the relative abundance of those populations which were positively connected with high metal resistant species increased in H group, and the same to their functional genes. It can be seen that OTU293 (*Verrucomicrobium*) was negatively connected with *Acidobacteria GP6* (highly resistant to heavy metals) and an unclassified OTU38, thus the intensity of genes derived from *Verrucomicrobium* decreased in H group, involved in starch (*amyA*) and hemicellules (*xylA*) degradation. By contrast, for OTUs negatively connected with microbial populations of high resistance, genes derived from them showed lower intensity in H group, such as OTU1109 and its functional genes, *amyA* and *xylA.* The result suggested that the abundance pattern of a kind of microbial population (or functional genes) is not only determined by whether they have metal resistance or not, but also the relationship with other populations.

In summary, response and adaptation of biological communities to environmental stress is a critical issue in ecology. In this study, we constructed a concept model that: part of microorganisms could take different strategies to survive under stress conditions, and other microorganisms would enhance their interactions with the former to adapt to the heavy metal contaminated environments, thus the relative abundance of these microorganisms increased while others decreased. Finally, a new microbial community with different composition formed. Therefore, microbial communities shifted their composition, functional structure, and network interaction to adapt to heavy metal contamination, and all these changes were significantly correlated with sediment properties.

## Materials and Methods

### Site description, sampling and DNA extraction

In this study, 16 sediment samples were collected from four sites in the Xiangjiang River (Table S1), China, with four samples in each site. These four sites had different distance from a sewage outlet. The composition of heavy metals including Hg, As, Co, Cd, Cr, Ni, Pb, Cu, Mn and Zn in the sediments was analyzed by ICP-AES^44^. Total sedimentary organic and N was quantified by Kjeldahl distillation^45^. The amount of total sedimentary organic C was analyzed by potassium dichromate oxidation-ferrous sulphate titrimetry^46^.

### DNA isolation, amplification, Illumina sequencing and data processing

DNA was extracted using a TIANamp Bacterial DNA Kit (MO BIO Laboratories, Inc., Carlsbad, CA). The V4 region of the 16S rRNA genes was amplified with the primer pair 515F (5′-GTGCCAGCMGCCGCGGTAA-3′) and 806R (5′- GGACTACHVGGGTWTCTAAT-3′) combined with Illumina adapter sequences, a pad and a linker of two bases, as well as barcodes on the reverse primers^47^. Sample libraries were generated from purified PCR products. The MiSeq 500 cycles kit was used for 2 × 250 bp paired-ends sequencing on MiSeq machine (Illumina, San Diego, CA).

Sequences with perfect matches to barcodes were split to sample libraries, and trimmed. OTU clustering was performed through UCLUST at 97% similarity level^48^, and taxonomic assignment was through the RDP classifier^49^ with a minimal 50% confidence estimate. The above steps were conducted through the Galaxy pipeline (http://zhoulab5.rccc.ou.edu/) developed by Qin *et al.* (unpublished). Subsequent analyses were performed in R^50^. Finally, samples were rarefied at 13,000 sequences per sample. All the 16S rRNA sequences were deposited in GenBank database and the accession number were KP784842 - KP788032.

### Microarray hybridization, data processing and statistical analysis

For each sample, microbial community DNA was extracted and purified as described previously^5^. Amplified DNA was labeled and hybridized with GeoChip 5.0. All GeoChip 5.0 hybridization data are available at the Institute for Environmental Genomics, University of Oklahoma (http://ieg.ou.edu/). The hybridized GeoChip 5.0 was analyzed as previously described^51^. Then functional gene diversity was calculated using Shannon-Weiner’s H′ and evenness. Statistical differences between the functional microbial communities from the different sites were analyzed by analysis of variance (ANOVA). Multivariate statistical analyses of GeoChip data included detrended correspondence analysis (DCA) for comparing the different functional gene communities and canonical correspondence analysis (CCA) for linking microbial communities to environmental variables. Mantel test^52^ was used to calculate correlations between functional gene abundance and environmental attributes. All other analyses were performed in R v. 2.6.1 with the packages vegan v. 1.11-3^50^.

### Network construction and characterization

16S rDNA sequencing data was used to construct phylogenetic molecular ecological networks (pMEN) as describe previously^53^. As previously described, random matrix theory (RMT) based approaches were used for network construction, hub and connector gene identification, and topological property determination with an automatic threshold^54^,^55^. To ensure correlation reliability, OTUs in at least 5 out of 8 replicates were used for network analysis. Various network properties such as average degree, average path distance, average clustering coefficient and modularity index were characterized. The network modules were generated using rapid greedy modularity optimization. The construction and major analyses of pMEN were performed online (http://ieg.ou.edu/). A stand T test was employed to determine the significance of network indexes between the pMENs and random networks and across different experimental conditions. Besides, based on singular value decomposition (SVD), eigengene network analysis was performed to summarize the gene abundance data from each module in pMENs. Finally, sample trait-based significance^13^ was defined, and a Mantel test was used to examine the relationships between the trait-based gene significance and sediment properties. The Cytoscape 2.6.0^56^ software was used to visualize the network graphs. Other information about genes (e.g., taxonomy, relative abundance), and edge information (e.g., weights and positive and negative correlations) was also imported into the software and visualized in the network figures. Since we were primarily interested in the impact of heavy metals on network interactions, the pMENs were constructed separately under low and high concentrations of heavy metals.

## Additional Information

**How to cite this article**: Yin, H. *et al.* An integrated insight into the response of sedimentary microbial communities to heavy metal contamination. *Sci. Rep.* **5**, 14266; doi: 10.1038/srep14266 (2015).

## Supplementary Material

Supplementary Information

Supplementary Dataset 1

## Figures and Tables

**Table 1:** Topological properties of the empirical pMENs of microbial communities in H group and L group.

Community	No. of original OTUs	Similarity threshold	Total nodes	Total links	R square of power-law	Average degree (avgK)	Average clustering coefficient (avgCC)	Average path distance (GD)	Module	Modularity
HighM	500	0.900	394	1609	0.873	8.168	0.324	3.598	24	0.617
LowM	268	0.900	183	406	0.824	4.437	0.364	4.449	17	0.711

**Table 2:** Mantel test of GeoChip data with environmental properties in each group.

Gene groups	Co	Cd	Cr	Ni	Mn	Zn	N	C
All	**0.028**	**0.021**	**0.023**	**0.021**	**0.03**	**0.035**	0.078	0.167
Carbon_Cycling	**0.032**	**0.018**	**0.026**	**0.033**	**0.017**	**0.035**	0.079	0.147
Metal_Homeostasis	0.056	**0.013**	**0.022**	**0.03**	**0.047**	0.053	0.091	0.154
Nitrogen	**0.027**	**0.012**	**0.025**	**0.031**	**0.012**	**0.035**	0.098	0.176
Organic_remediation	0.08	0.077	**0.014**	**0.03**	0.072	0.062	**0.043**	**0.044**
Other	0.097	**0.041**	**0.039**	**0.05**	**0.035**	**0.048**	0.087	0.063
Phosphorus	**0.016**	**0.02**	**0.025**	**0.028**	**0.019**	**0.027**	0.055	0.125
Sulfur	**0.024**	**0.015**	**0.017**	**0.016**	**0.02**	**0.028**	0.076	0.143
Virulence	0.079	**0.034**	0.058	0.064	**0.047**	0.05	0.164	0.226

*Significant differences (P < 0.05) are indicated in bold.
