# Supplementary material for: An integrated insight into the response of sedimentary microbial communities to heavy metal contamination
Source: Sci Rep. 2015 Sep 22;5:14266. doi: 10.1038/srep14266 (PMC4585741; doi:10.1038/srep14266)
Supplement: Supplementary Information [file srep14266-s1.pdf]

An integrated insight into the response of sedimentary microbial communities to heavy metal contamination (Supplementary information)

Huaqun Yin, Jiaojiao Niu, Youhua Ren, Jing Cong, Xiaoxia Zhang, Fenliang Fan, Yunhua Xiao, Xian Zhang, Jie Deng, Ming Xie, Zhili He, Jizhong Zhou, Yili Liang, Xueduan Liu

Authors:
Huaqun Yin (yinhuaqun@gmail.com)
Youhua Ren (renyohua@163.com)
Jiaojiao Niu (135611006@csu.edu.cn)
Jing Cong (yqdh77@163.com)
Xiaoxia Zhang (zhangxiaoxia@caas.cn)
Fenliang Fan (fanfenliang@caas.cn)
Yunhua Xiao (huazipiaoling.123@163.com)
Xian Zhang (zixuange2010@126.com)
Jie Deng (Jiedeng@ou.edu)
Ming Xie (Ming.Xie-1@ou.edu)
Zhili He (zhili.he@ou.edu)
Jizhong Zhou (jzhou@ou.edu)
Yili Liang (liangyili@hotmail.com)
Xueduan Liu (xueduanliu@yahoo.com)

Figure S1. Detrended correspondence analysis (DCA) of sediment properties.

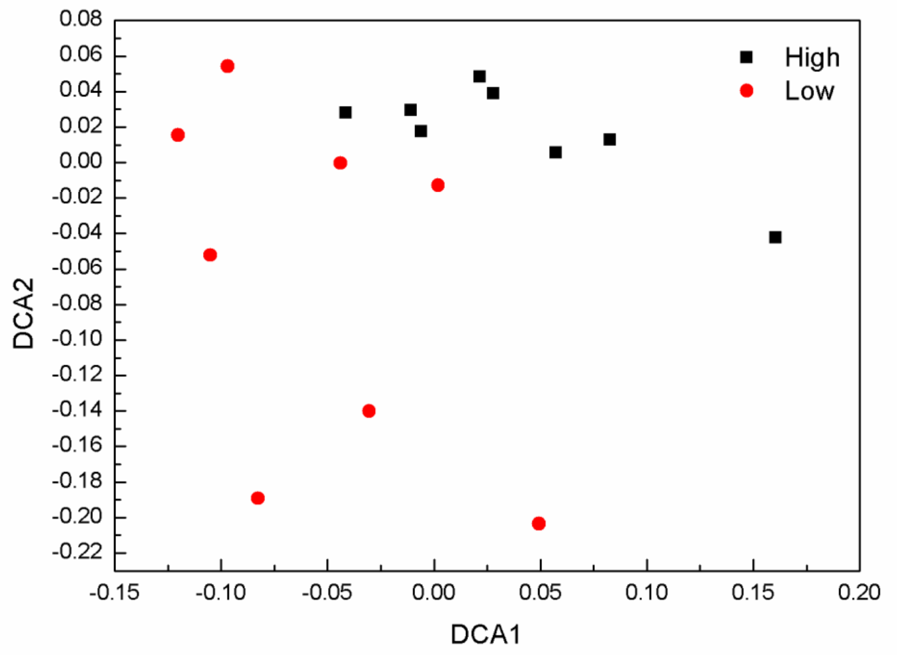

Figure S2. Rarefaction of 16S rRNA gene sequencing in each group.

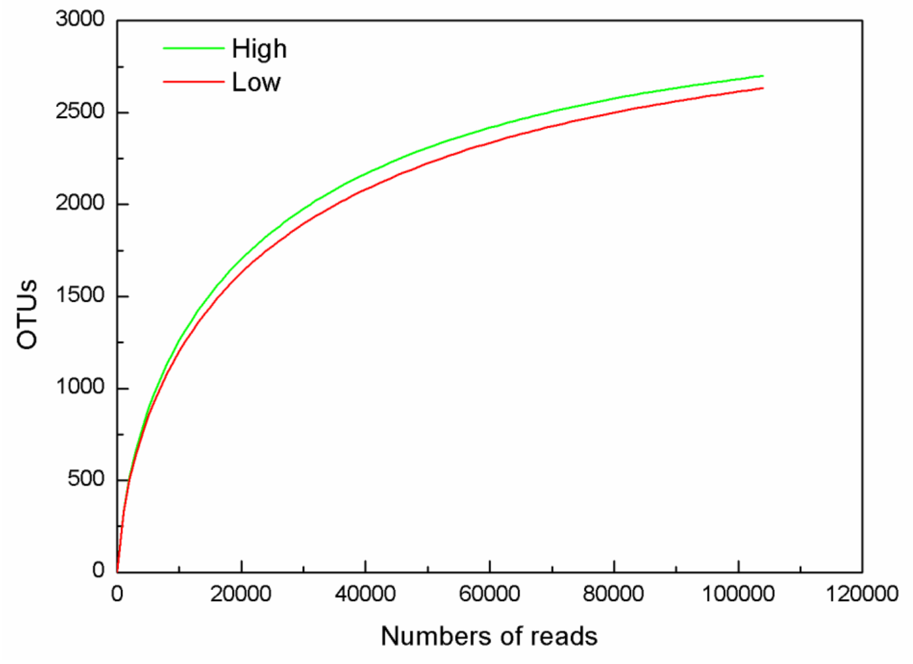

Figure S3. Percentage of each phylum in H group and L group. Only four important phyla were labeled with percentage, including *Proteobacteria*, *Firmicutes*, *Chloroflexi*, and *Actinobacteria*.

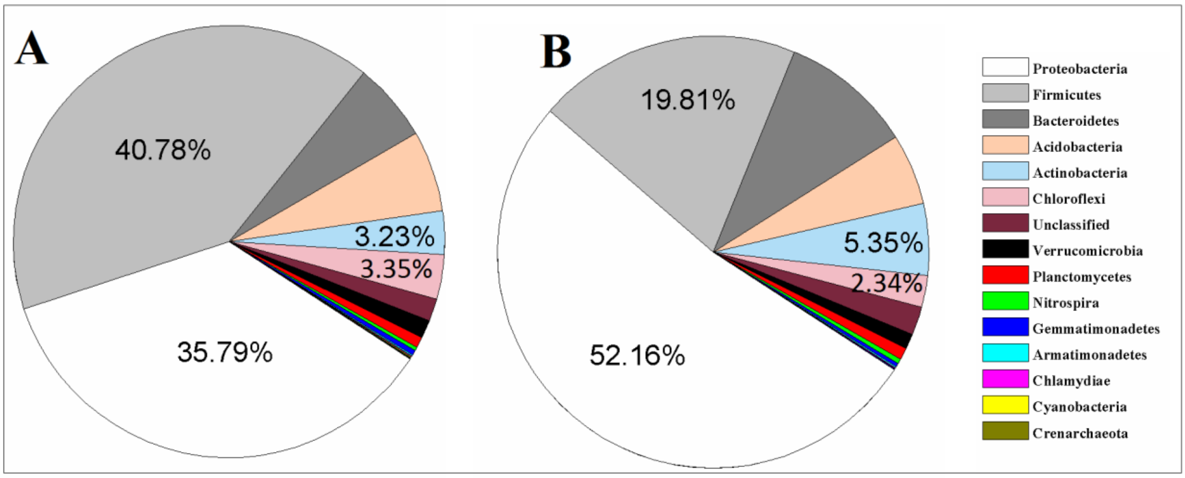

Figure S4. Scatter plots showing the fittings of the OTU connectivity distributions of the pMENs in H and L group. The x axis is the node connectivity (k). The y axis is the number of nodes under a given connectivity. The values on both axes were log transformed. Lines and R^2 values show the best fit of the data to the model. (A) pMEN of H group. (B) pMEN of L group.

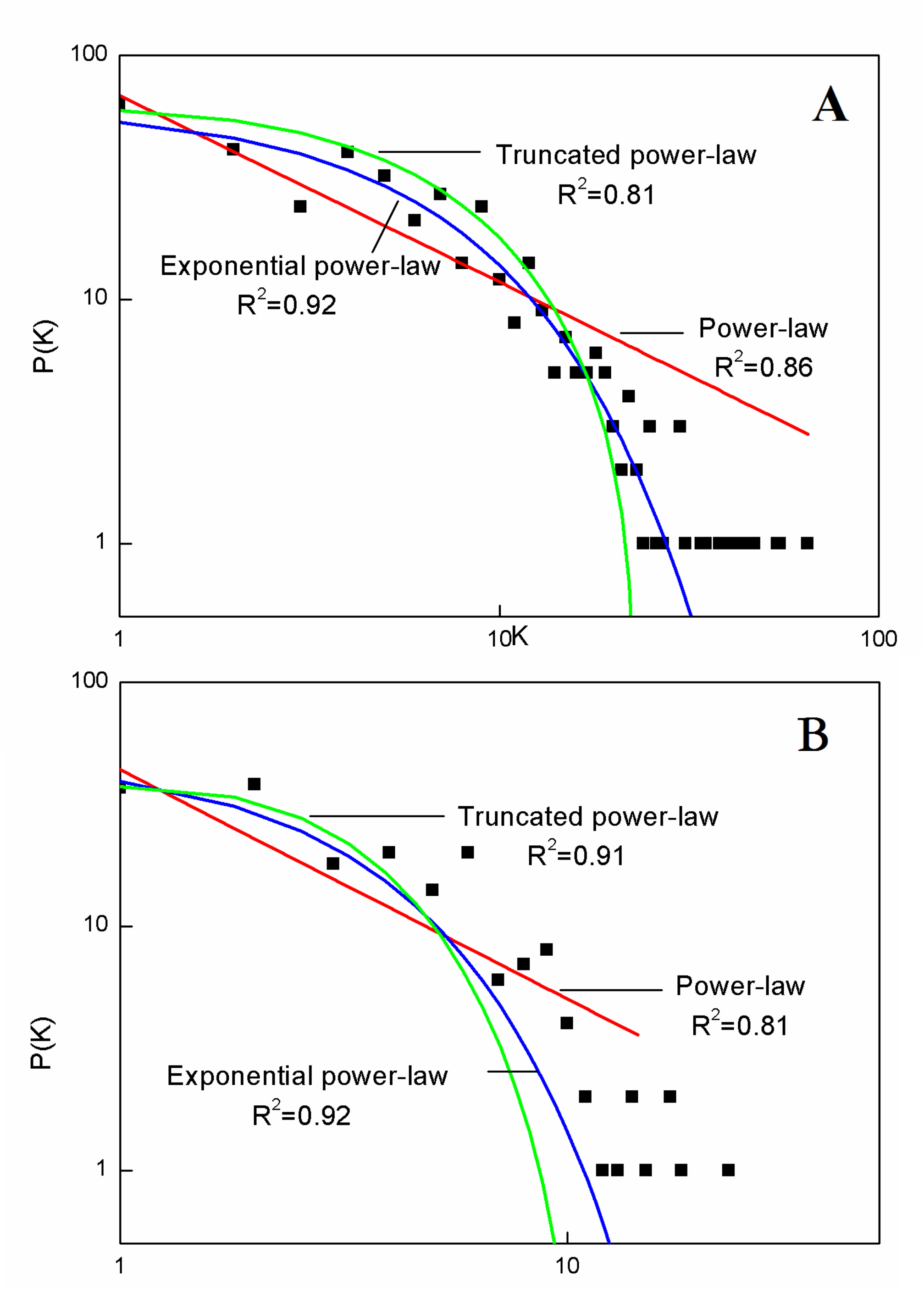

Figure S5. Modular organization of the pMENs with 16S rRNA gene-based metagenomic data. The networks were constructed by the RMT-based approach with the pyrosequencing data from H group (A) (8 samples) and L group (C) (8 samples). Colors of the nodes indicate different major phyla. Clear modular architecture was observed in this pMEN. Each node signifies an OTU which could correspond to a microbial population. Colors of the nodes indicate different major phyla. A blue line indicates a positive interaction between two individual nodes, while a red line indicates a negative interaction. The numbers indicate different modules or sub-modules determined by the fast greedy modularity optimization method. All data show that the phylogenetic MENs have a modular architecture. The sizes of individual modules are plotted in panels B (H group) and D (L group)

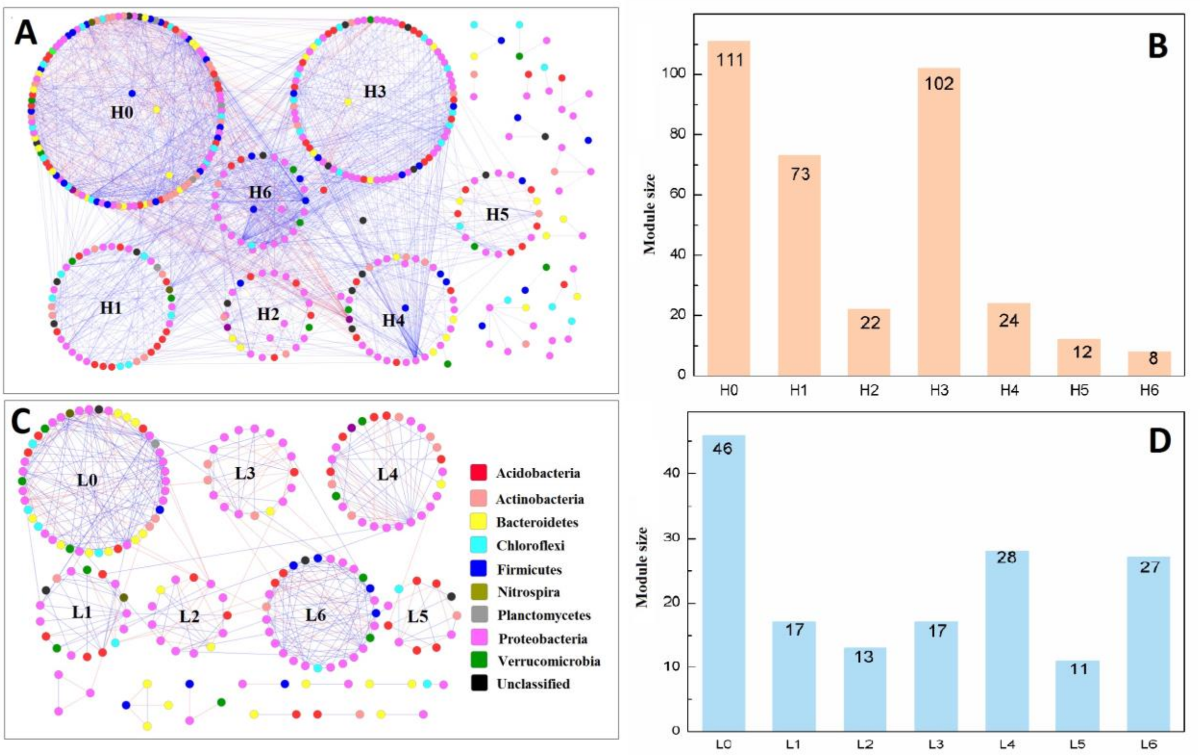

Figure S6. Example of eigengene network analysis with module E5 in H group. (A) Heat map of the standardized relative abundance (SRA) of OTUs across different samples. Rows correspond to individual OTUs in the module, whereas columns are the samples. Red corresponds to the OTUs whose SRAs are >0, and green signifies those whose SRAs are >0. (B) SRA of the corresponding eigengene across the samples. The parameter Φ indicates the percentage of the total variance explained by the eigengene. (C) Module memberships identify groups of OTUs that consistently coexist in these microbial communities. Only 5 OTUs with significant module memberships are shown here. The values in parentheses are module memberships. (D) Module visualization showing the interactions among different OTUs within the module. Blue line, positive correlation; red line, negative correlation. The different colors of the shading of nodes represent different phylogenetic groups. (E) Mantel test of eigengenes with sediment properties of module E5.

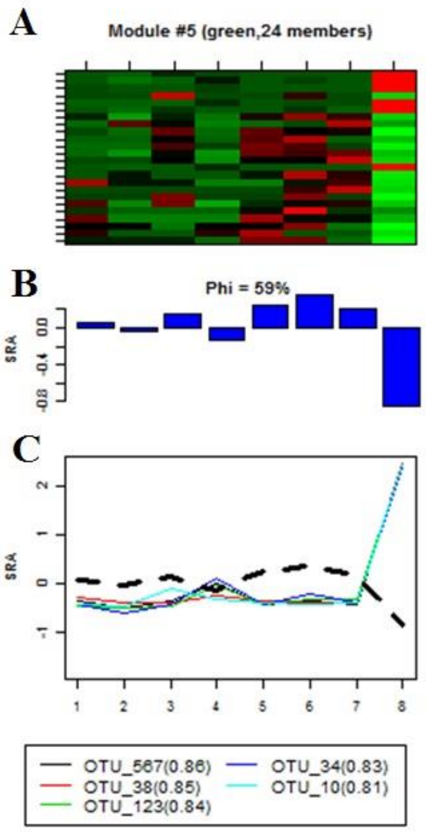

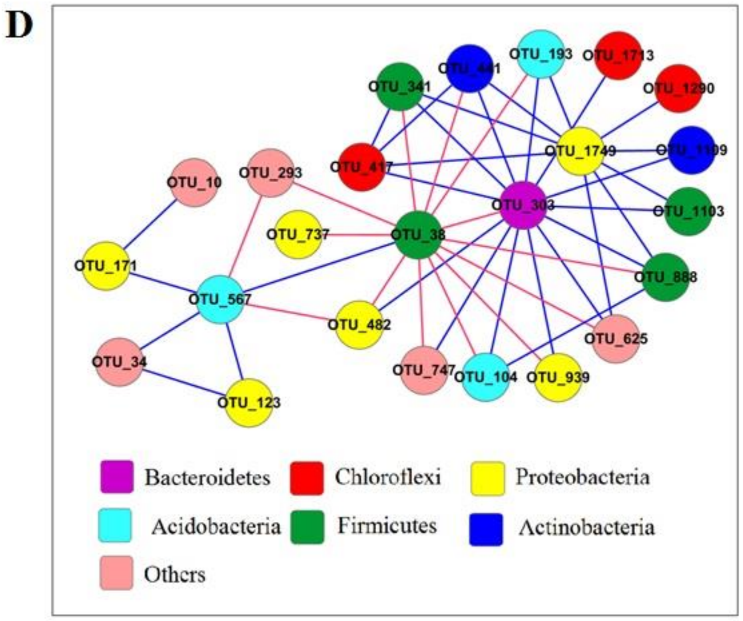

E

	Hg	Cr	Pb	Cu	Zn	C
r	-0.9	-0.57	-0.66	-0.57	-0.61	-0.73
p	0.003	0.1	0.07	0.1	0.1	0.04

Table S1. Location and altitude of sampling sites.

Samples	East	North	Altitude (m)
A1	112°59'40.2432″	27°58'51.1140″	31.3
A2	112°59'36.9960″	27°58'56.0568″	37.3
A3	112°59'36.9600″	27°58'58.2060″	32.7
A4	112°59'35.4480″	27°58'59.8008″	30.7
B1	112°59'26.6532″	27°58'50.2608″	32.7
B2	112°59'27.6288″	27°58'51.5532″	28.5
B3	112°59'27.6612″	27°58'52.6368″	33.5
B4	112°59'21.9228″	27°58'59.0700″	35.9
C1	112°59'26.6352″	27°58'45.8832″	31.3
C2	112°59'26.1492″	27°58'47.6472″	28.7
C3	112°59'25.1988″	27°58'50.3472″	31.7
C4	112°59'24.9000″	27°58'51.1788″	27.6
D1	112°59'12.7428″	27°58'33.4992″	28.1
D2	112°59'8.4912″	27°58'35.6268″	28.8
D3	112°59'4.5240″	27°58'38.2800″	29.3
D4	112°58'56.8848″	27°58'39.5688″	29.5

Table S2. Sediment properties and ANOVA analysis between two groups.

	Hg	As	Co	Cd	Cr	Ni	Pb	Cu	Mn	Zn	N	C
H1	0.66	327	27.9	22.4	92	60.3	151	78	1661	535	1.87	19.2
H2	0.69	362	30.8	27.7	98	65.7	160	76	3204	627	1.67	17.4
H3	0.69	204	18.2	23.9	88	49.9	105	75	1678	454	1.37	16.4
H4	0.56	99	15.7	14.2	71	38.9	80	49	1507	370	1.13	13.7
H5	0.39	222	14.9	12.6	69	36.2	81	53	1527	335	0.7	12.2
H6	0.52	232	17.3	16.5	71	42.7	96	63	2026	398	1.04	14.7
H7	0.52	138	14.5	11.1	57	31.9	78	44	1497	303	0.7	13
H8	1.1	247	19.5	21.8	98	57.2	175	89	2593	607	1.49	21.6
L1	0.51	84	17.2	16.5	75	44.6	106	57	1426	422	1.03	13.5
L2	0.15	62	8.2	3.3	46	19.3	94	34	1306	238	0.13	4.66
L3	0.17	33	6.5	1.6	36	14.4	77	27	700	179	0.05	2.58
L4	0.65	104	19.3	28.2	89	52.5	136	81	2473	544	1.17	13.5
L5	0.18	71	10.3	0.6	57	28.7	68	31	536	124	0.63	9.15
L6	0.24	113	12.6	6.9	57	32	66	37	1124	212	0.77	8.35
L7	0.16	66	12.6	2.7	56	31.4	152	38	890	177	0.94	12.5
L8	0.14	42	12.7	1.8	57	28.7	47	29	604	120	0.94	12.5
P value	**0.006**	**0.004**	**0.028**	0.061	**0.032**	**0.032**	0.248	**0.039**	**0.026**	**0.033**	**0.035**	**0.01**

*All values are in ppm.

*Significant differences (P<0.05) are indicated in bold.

Table S3(a). Alpha diversity of 16S rDNA sequencing data and dissimilarity test between two groups

Gene	Alpha diversity		Dissimilarity test	
	Shannon Index	Pielou evenness	Mean distance	Significance
High	10.0415475	0.999380625	0.131	0.107
Low	10.06584	0.999376838		

Table S3(b). Alpha diversity of GeoChip data and dissimilarity test between two groups

OTU	Alpha diversity		Dissimilarity test	
	Shannon Index	Pielou evenness	Mean distance	Significance
High	4.2267795	0.621360063	0.537	0.0509
Low	4.14525925	0.6416976		

Table S4. Mantel test of 16S rDNA sequencing data with sediment properties.

Phylum	Hg	As	Cd	Cr	Cu	Mn	Zn	TN	TC
Whole	**0.019**	0.09	**0.02**	0.119	**0.028**	**0.023**	**0.007**	0.429	0.139
Actinobacteria	0.111	0.219	**0.019**	0.327	0.101	0.068	**0.021**	0.275	0.22
Chlorobi	0.182	**0.018**	0.295	0.443	0.151	0.326	0.362	0.498	0.449
Deinococcus-Thermus	0.312	0.219	0.222	**0.01**	0.163	0.384	0.481	**0.005**	**0.002**
Firmicutes	**0.034**	**0.034**	**0.016**	0.086	**0.033**	**0.011**	**0.01**	0.276	0.207
Nitrospira	0.272	0.197	0.316	**0.031**	0.14	0.37	0.434	**0.033**	**0.022**
Planctomycetes	0.476	0.267	0.09	**0.007**	0.144	0.113	0.266	**0.027**	**0.016**
Proteobacteria	**0.023**	0.41	**0.03**	0.169	**0.036**	0.101	**0.018**	0.657	0.181
Verrucomicrobia	0.36	0.554	**0.047**	0.062	0.134	0.139	0.155	0.101	0.064
WS3	0.084	**0.021**	0.433	0.3	0.161	0.072	0.146	0.461	0.304

*Abbreviations: TN, total nitrogen; TC, total carbon.

*Significant differences (P<0.05) are indicated in bold.

Table S5. Difference of functional gene in each group as examined by dissimilarity test.

	Distance	Significance
carbon	46.774	**0.0529**
nitrogen	25.336	**0.0539**
metal	18.944	**0.0619**
phosphrus	14.275	**0.0589**
sulfure	21.126	**0.0529**
organic	31.745	**0.0699**
secondary	3.044	0.592
virulence	8.753	0.108

*Significant differences (P<0.05) are indicated in bold.

Table S6(a). Normalized gene intensity and category of genes significantly more abundant in H group.

High	Low	p value	Gene	Gene_category	Subcategory1	Subcategory2
82.61491	78.19641	**	limeh	Carbon Cycling	Carbon degradation	Terpenes
151.2909	147.7333	**	ccmL	Carbon Cycling	Carbon fixation	Bacterial Microcompartments
124.8928	121.5991	**	FBP_aldolase	Carbon Cycling	Carbon degradation	Pectin
33.40419	30.53041	**	alpha_galactosidase_fungi	Carbon Cycling	Carbon degradation	Other
159.5686	157.4446	**	vana	Carbon Cycling	Carbon degradation	Vanillin/Lignin
249.1995	247.2205	**	cutinase	Carbon Cycling	Carbon degradation	Cutin
6.986259	5.613651	**	lmo	Carbon Cycling	Carbon degradation	Terpenes
5.915004	4.668454	**	ccmN	Carbon Cycling	Carbon fixation	Bacterial Microcompartments
71.28013	70.6639	*	protease_serine_fungi	Carbon Cycling	Carbon degradation	protein
3.146143	2.753303	**	mcl	Carbon Cycling	Carbon fixation	3-hydroxypropionate bicycle
1.08186	0.765011	*	C_CoA_hydratase	Carbon Cycling	Carbon fixation	3-hydroxypropionate/4-hydroxybutyrate cycle
215.4075	215.1466	**	TIM	Carbon Cycling	Carbon fixation	Calvin cycle
3.601192	3.550444	*	4hbcd_dic4hb	Carbon Cycling	Carbon fixation	Dicarboxylate/4-hydroxybutyrate cycle
9.848407	9.821527	**	PEPC	Carbon Cycling	Carbon fixation	Dicarboxylate/4-hydroxybutyrate cycle
2.924859	2.900958	**	lipase_fungi	Carbon Cycling	Carbon degradation	Lipids
2.700552	2.67896	**	fumarase_3HP4HB	Carbon Cycling	Carbon fixation	Reductive acetyl–CoA pathway
2.633423	2.613423	**	mcr	Carbon Cycling	Carbon fixation	3-hydroxypropionate bicycle
1.544033	1.529435	**	mct	Carbon Cycling	Carbon fixation	3-hydroxypropionate bicycle
2.028495	2.018401	**	4hbcd	Carbon Cycling	Carbon fixation	3-hydroxypropionate/4-hydroxybutyrate cycle
5.058804	5.051935	**	suc_CoA_red_DiC4HB	Carbon Cycling	Carbon fixation	Dicarboxylate/4-hydroxybutyrate cycle
1.120328	1.119081	**	sdhA	Carbon Cycling	Carbon fixation	3-hydroxypropionate bicycle
29.00072	27.74852	*	chrr	Metal Homeostasis	Chromium	Detoxification
15.349	14.68666	*	metc	Metal Homeostasis	Mercury	Detoxification
71.66029	71.41566	**	merb	Metal Homeostasis	Mercury	Detoxification
1.236746	1.23331	**	silaffin	Metal Homeostasis	Silicon	Biosynthesis
729.3642	724.39	**	narg	Nitrogen	Denitrification	NA
403.6078	399.9004	**	urec	Nitrogen	Ammonification	NA
96.3308	95.81379	**	nasa	Nitrogen	Assimilatory N reduction	NA
167.3765	163.5685	*	badh	Organic Remediation	Aromatics	Other aromatics
14.846	12.77264	**	xyll	Organic Remediation	Aromatics	Aromatic carboxylic acid
48.32891	46.61159	**	bend	Organic Remediation	Aromatics	Aromatic carboxylic acid
114.5375	113.0018	**	nhh	Organic Remediation	Aromatics	Nitoaromatics
242.021	240.7758	**	poba	Organic Remediation	Aromatics	Aromatic carboxylic acid
142.3664	141.177	**	cmci	Organic Remediation	Aromatics	Other aromatics
20.75604	19.64767	*	chnd	Organic Remediation	Other Hydrocarbons	NA
18.75319	17.75345	*	pchcf	Organic Remediation	Aromatics	BTEX and related aromatics
12.71203	11.75408	**	nahf	Organic Remediation	Aromatics	Polycyclic aromatics
4.794441	4.038268	*	msad	Organic Remediation	Others	NA
2.879194	2.225463	*	cbaa	Organic Remediation	Aromatics	Chlorinated aromatics
88.18014	87.60819	*	one_ring_12diox	Organic Remediation	Aromatics	Other aromatics
0.804356	0.336862	*	dbdc	Organic Remediation	Aromatics	Heterocyclic aromatics

1.092286	0.667919	*	nahc_2	Organic Remediation	Aromatics	Polycyclic aromatics
45.62803	45.40861	*	nfsa_2	Organic Remediation	Aromatics	Nitoaromatics
13.59835	13.5385	**	bco	Organic Remediation	Aromatics	Aromatic carboxylic acid
99.58326	99.53223	**	mult_ring_12DiOx	Organic Remediation	Aromatics	Other aromatics
4.02189	3.978148	**	bbs	Organic Remediation	Aromatics	BTEX and related aromatics
2.816317	2.774689	**	mhqa	Organic Remediation	Aromatics	Nitoaromatics
2.718511	2.681181	**	nbzc	Organic Remediation	Aromatics	Nitoaromatics
3.775855	3.753185	**	bbsg	Organic Remediation	Aromatics	BTEX and related aromatics
2.649245	2.631612	**	cdo	Organic Remediation	Aromatics	Other aromatics
1.145989	1.137885	**	nbza	Organic Remediation	Aromatics	Nitoaromatics
2.554149	2.547293	**	nbzb	Organic Remediation	Aromatics	Nitoaromatics
178.4118	175.6465	**	soxY	Sulfur	Sulfur Oxidation	NA
282.1989	280.7127	**	cysJ	Sulfur	Reduction	NA

Table S6(b). Normalized gene intensity and category of genes significantly less abundant in H group.

High	Low	p value	Gene	Gene_category	Subcategory1	Subcategory2
1.179592	1.214678	*	dextranase_fungi	Carbon Cycling	Carbon degradation	sucrose
43.04822	43.10139	**	mnp	Carbon Cycling	Carbon degradation	Lignin
3.672285	3.746374	**	ccl	Carbon Cycling	Carbon fixation	Reductive tricarboxylic acid cycle
8.169744	8.265637	*	sucA_DiC4HB	Carbon Cycling	Carbon fixation	Dicarboxylate/4-hydroxybutyrate cycle
177.4958	177.6416	*	endoglucanase	Carbon Cycling	Carbon degradation	Chitin
7.336763	7.583725	**	oorA	Carbon Cycling	Carbon fixation	Reductive tricarboxylic acid cycle
23.38332	24.08689	**	mmox	Carbon Cycling	Methane	Methane oxidation
432.225	433.3116	**	tktA	Carbon Cycling	Carbon fixation	Calvin cycle
17.36115	18.50949	*	Glucose_oxidase_fungi	Carbon Cycling	Carbon degradation	glucose
15.18694	16.64461	**	chitin_deacetylase_fungi	Carbon Cycling	Carbon degradation	Chitin
131.9039	133.8049	**	glucoamylase	Carbon Cycling	Carbon degradation	Starch
47.27027	49.23928	**	exopolygalacturonase_fungi	Carbon Cycling	Carbon degradation	Cellulose
313.395	315.6831	**	cellobiase	Carbon Cycling	Carbon degradation	Cellulose
94.97057	97.69419	**	pula	Carbon Cycling	Carbon degradation	Starch
242.8304	246.1505	**	GAPDH_Calvin	Carbon Cycling	Carbon fixation	Calvin cycle
623.4501	629.7217	*	chitinase	Carbon Cycling	Carbon degradation	Chitin
302.2232	312.5634	**	phenol_oxidase	Carbon Cycling	Carbon degradation	Lignin
12.41665	12.96206	**	silicon_transporter	Metal Homeostasis	Silicon	Transport
9.168263	9.236702	**	hzo	Nitrogen	Anammox	NA
339.3049	339.8729	**	nirs	Nitrogen	Denitrification	NA
38.87932	39.51393	**	narb	Nitrogen	Assimilatory N reduction	NA
25.59453	27.87074	**	hao	Nitrogen	Nitrification	NA
558.6419	580.1362	**	nifh	Nitrogen	Nitrogen fixation	NA
52.90932	52.93365	**	amie	Organic Remediation	Aromatics	Other aromatics
2.618172	2.647529	**	onr	Organic Remediation	Others	NA
9.884595	9.934663	**	alkj	Organic Remediation	Other Hydrocarbons	NA
10.09031	10.19036	**	chnc	Organic Remediation	Other Hydrocarbons	NA

7.622455	7.754502	**	mauab	Organic Remediation	Herbicides related compound	NA
116.3534	116.4891	**	xylg	Organic Remediation	Aromatics	Aromatic carboxylic acid
53.58515	53.73191	*	p450aro	Organic Remediation	Aromatics	polycyclic aromatics
3.933702	4.085466	**	nahb	Organic Remediation	Aromatics	Polycyclic aromatics
5.153194	5.402347	*	msmabcd	Organic Remediation	Others	NA
12.18295	12.46649	*	scnabc	Organic Remediation	Others	NA
10.22258	10.52724	*	phdk	Organic Remediation	Polycyclic aromatics	NA
62.30123	62.61557	**	mhpa	Organic Remediation	Aromatics	Aromatic carboxylic acid
1.222383	1.633192	**	thmAB	Organic Remediation	Others	NA
3.933827	4.577882	**	cpne	Organic Remediation	Other Hydrocarbons	NA
13.29744	13.99033	*	cara	Organic Remediation	Aromatics	Heterocyclic aromatics
14.72722	15.47769	**	phta	Organic Remediation	Aromatics	Aromatic carboxylic acid
80.8987	81.75181	**	atza	Organic Remediation	Herbicides related compound	NA
83.49629	84.51716	**	nagg	Organic Remediation	Aromatics	Aromatic carboxylic acid
6.900089	7.952006	**	cpnc	Organic Remediation	Other Hydrocarbons	NA
56.73122	58.27488	*	linc	Organic Remediation	Pesticides related compound	NA
149.4188	151.3722	**	arylest	Organic Remediation	Aromatics	Other aromatics
105.0694	108.7868	**	bphc	Organic Remediation	Aromatics	Polycyclic aromatics
172.9551	178.1853	**	pcag	Organic Remediation	Aromatics	Other aromatics
280.3449	285.2487	**	ppk	Phosphorus	Polyphosphate synthesis	NA
422.1715	429.5132	*	dsrB	Sulfur	sulfite reduction	NA
563.3559	574.2286	**	dsra	Sulfur	sulfite reduction	NA
121.0414	121.1585	*	ben_bcla	virulence	NA	NA
3.236346	3.374337	**	fosx	Virulence	Antibiotic resistance	Degradation
28.48364	33.44597	**	phospholipase_B_fungi	virulence	degradation	NA

*Only Significant differences (P<0.1) are showed. *p<0.1, **p<0.05

Table S7. Mantel test of Geochip data with environmental attributes.

Gene	As	Co	Cd	Cr	Ni	Pb	Cu	Mn	Zn	N	C
Whole	0.173	**0.027**	**0.013**	**0.029**	**0.022**	0.604	0.075	**0.018**	**0.022**	0.061	0.157
FBP_aldolase	0.079	**0.006**	**0.02**	**0.034**	**0.025**	0.381	0.1	**0.005**	**0.028**	0.071	0.151
SoxA	0.207	0.059	**0.018**	**0.032**	**0.047**	0.631	0.15	**0.049**	**0.045**	0.141	0.141
SoxB	0.103	**0.02**	**0.007**	**0.018**	**0.012**	0.496	0.057	**0.005**	**0.021**	0.088	0.172
SoxC	0.428	0.067	**0.015**	**0.001**	**0.009**	0.621	**0.043**	0.125	0.108	**0.014**	**0.017**
SoxV	0.071	**0.024**	**0.037**	**0.015**	**0.013**	0.588	0.116	**0.01**	**0.05**	0.059	0.085
acetylglucosaminidase	0.094	**0.038**	**0.02**	**0.044**	**0.038**	0.517	0.1	**0.01**	**0.022**	0.129	0.186
alkb	0.184	0.061	**0.019**	**0.012**	**0.015**	0.707	0.08	**0.049**	0.053	**0.049**	0.13
aoxb	0.231	**0.019**	**0.004**	**0.017**	**0.01**	0.504	**0.043**	**0.02**	**0.01**	0.056	0.054
arra	0.407	0.084	**0.048**	**0.023**	**0.033**	0.9	0.168	0.117	0.132	0.057	0.101
arsc	0.243	0.055	**0.031**	**0.036**	**0.034**	0.714	0.156	**0.045**	0.072	0.101	0.209
arsm	0.428	0.161	**0.01**	0.064	0.106	0.676	0.164	**0.025**	0.083	0.324	0.356
arylest	0.122	**0.015**	**0.013**	**0.013**	**0.011**	0.712	0.058	**0.046**	**0.032**	**0.043**	0.129
cueo	0.059	**0.011**	**0.006**	**0.02**	**0.02**	0.705	0.081	**0.015**	**0.02**	0.063	0.169
dsrB	0.117	**0.03**	**0.009**	**0.023**	**0.007**	0.695	0.087	**0.039**	**0.036**	0.085	0.164
dsra	0.146	**0.027**	**0.028**	**0.017**	**0.015**	0.632	0.061	**0.014**	**0.027**	**0.049**	0.084
metc	**0.038**	**0.017**	**0.047**	0.126	0.052	**0.017**	0.076	**0.005**	**0.032**	0.074	0.255
nirk	0.153	**0.039**	**0.017**	**0.034**	**0.029**	0.714	0.131	**0.021**	**0.022**	0.14	0.311
nirs	0.167	0.057	**0.019**	**0.03**	**0.036**	0.624	0.1	**0.02**	**0.031**	0.116	0.223
ppk	0.147	**0.017**	**0.013**	**0.02**	**0.028**	0.508	0.068	**0.023**	**0.018**	0.058	0.139
ppx	0.126	**0.025**	**0.013**	**0.034**	**0.015**	0.523	0.083	**0.015**	**0.025**	0.071	0.169
silicon_transporter	0.088	**0.037**	**0.008**	**0.014**	**0.015**	0.652	**0.033**	**0.03**	**0.019**	**0.034**	**0.023**
soxY	0.228	**0.049**	**0.01**	**0.017**	**0.018**	0.597	0.081	**0.03**	0.051	0.081	0.138
tehb	0.387	0.078	**0.036**	**0.05**	0.069	0.623	0.204	0.058	0.073	0.18	0.374

*Significant differences ($P<0.05$) are indicated in bold.
